# Expression Profile of Transfer RNA-Derived Fragments in Chinese Mitten Crab (*Eriocheir sinensis*) After Challenge with *Aeromonas hydrophila*

**DOI:** 10.3390/ani16203312

**Published:** 2026-10-21

**Authors:** Tongtong Kong, Lei Liu, Hanyu Liu, Ngoc Tuan Tran

**Affiliations:** 1 School of Life Sciences, Qufu Normal University, Qufu 273165, China; 2 Shandong Key Laboratory of Wetland Ecology and Biodiversity Conservation in the Lower Yellow River, Qufu 273165, China; 3 Guangdong Provincial Key Laboratory of Marine Biology, Shantou University, Shantou 515063, China; 4 Institute of Marine Sciences, Shantou University, Shantou 515063, China

**Keywords:** Chinese mitten crab, *Aeromonas hydrophila*, tRNA-derived fragments, immune response

## Abstract

The Chinese mitten crab (*Eriocheir sinensis*) is an important cultured crab in China; however, its aquaculture productivity is severely limited by bacterial pathogens, including *Aeromonas hydrophila*. Transfer RNA-derived fragments (tRFs) are an emerging class of functional small non-coding RNAs that have been implicated in host–pathogen interactions and immune regulation; yet, their roles in the antibacterial defense of crustaceans remain poorly understood. To investigate the immune response of the Chinese mitten crab to *A. hydrophila* infection, we employed high-throughput sequencing to characterize the expression profiles and functional features of tRFs in crab hemocytes following a bacterial challenge. The results revealed that tRFs exhibited a conserved length distribution (15–20 nt) and subtype composition, with 5′-tRFs representing the dominant subtype in both control and infected groups. Notably, infection specifically induced the abundance of tRF fragments of 35-nt tRF fragments and slightly altered the proportion of tRF subtypes. Differential expression analysis identified 415 significantly upregulated and 3 downregulated tRFs upon *A. hydrophila* infection, suggesting widespread changes in tRF abundance during infection. Target prediction and functional enrichment analyses were performed for the top 30 differentially expressed tRFs; the predicted targets were significantly associated with VEGF signaling pathway, DNA replication proteins, FoxO signaling pathway, and NF-kappa B signaling pathway. The reliability of the sequencing data was confirmed by stem-loop RT-qPCR validation. Collectively, our findings advance the understanding of tRF expression in crustacean immunity and suggest that tRFs may be associated with the immune response of crabs against bacterial infection, pending functional validation.

## Simple Summary

The Chinese mitten crab (*Eriocheir sinensis*) is a commercially vital aquaculture species. However, *Aeromonas hydrophila* infection poses a serious economic threat to the aquaculture industry. Elucidating the antibacterial immune response of the Chinese mitten crab is thus of critical importance. In this study, we characterized the expression profiles and functional features of transfer RNA-derived fragments (tRFs) in crab hemocytes following a bacterial challenge. We found that *A. hydrophila* infection triggered a specific and robust reprogramming of the tRF repertoire, which likely regulated the host innate immune responses and metabolic stability during infection. Our findings provide basic information on the function of tRFs as potential non-coding RNA biomarkers during bacterial infection, serving as a useful basis for future studies on disease prevention and control in crab farming.

## 1. Introduction

The Chinese mitten crab (*Eriocheir sinensis*) is an important crab species with significant economic value in both domestic Chinese and international markets [1]. However, bacterial diseases represent one of the most critical limiting factors in crab aquaculture, causing massive economic losses and threatening the sustainable development of the industry [2]. In 2022, disease-related losses in Chinese mitten crab production amounted to RMB 6.6 billion [3]. Among these pathogens, the Gram-negative bacterium *Aeromonas hydrophila* is a ubiquitous opportunistic pathogen that induces severe hemorrhagic septicemia and tissue damage in cultured Chinese mitten crabs [4,5]. Previous studies have shown that *A. hydrophila* infection induces oxidative stress and inflammatory and immune responses in Chinese mitten crabs, and also alters the mRNA expression of apoptosis-related genes [4]. *A. hydrophila* infection can cause high mortality in cultured animals, and its widespread distribution and rapid transmission pose a serious economic threat to the aquaculture industry [2,5]. Given these challenges, elucidating the antibacterial immune response of the Chinese mitten crab is of critical importance. Like other invertebrates, Chinese mitten crabs lack adaptive immunity and rely entirely on innate immune defenses, including hemocyte-mediated immune responses, inflammatory signaling cascades, and metabolic detoxification pathways, to combat pathogenic invasion [6]. Although previous transcriptomic studies have extensively characterized immune-related genes and non-coding RNAs, including microRNAs, mRNAs, long non-coding RNAs, and circular RNAs, involved in antimicrobial responses in Chinese mitten crabs [7,8,9], the regulatory functions of small non-coding RNA (sncRNA) subtypes, especially for emerging functional sncRNAs such as transfer RNA-derived fragments (tRFs), remain poorly understood.

tRFs constitute a novel class of small non-coding RNAs generated from mature or precursor tRNAs, encompassing 5′-tRFs, 3′-tRFs, and i-tRFs [10]. Previous studies have confirmed that tRFs participate in multiple biological processes through both post-transcriptional and post-translational mechanisms [11,12,13]. At the transcript level, tRFs can modulate mRNA stability by displacing RNA-binding proteins. For example, tRF-24 binds CELF1, shielding it from AKT1-mediated phosphorylation and driving its nuclear translocation, thereby promoting esophageal squamous cell carcinoma progression [14,15]. At the protein level, tRFs directly regulate protein stability and activity. Specifically, tRF-22 binds hnRNPAB to prevent its ubiquitination-mediated degradation [10], while tRF-GlyGCC acts as a scaffold to facilitate the FGFR1-mediated phosphorylation of LDHA, thereby enhancing its enzymatic activity [16]. Notably, a single tRF can even regulate the expression or stability of its target molecules via two distinct intracellular mechanisms [10,13]. Collectively, these findings suggest that tRFs may play an important role in regulating host immunity. Nevertheless, the expression profiles and functional roles of tRFs in crustaceans (including the Chinese mitten crab) in response to pathogenic challenge have not been thoroughly studied.

To address these research gaps, the present study performed high-throughput tRF sequencing to profile genome-wide changes in tRF expression in the hemocytes of Chinese mitten crab after *A. hydrophila* infection. The analysis characterized the basic structural features of tRFs, identified differentially expressed tRFs (DE-tRFs), and explored their potential biological functions through GO and KEGG enrichment analyses. Furthermore, stem-loop RT-qPCR was employed to validate the accuracy and reliability of the sequencing data. Our findings provide basic information on the function of tRFs as novel non-coding RNA biomarkers under bacterial infection, contributing to a broader understanding of the immune response mechanisms in crabs. This knowledge will inform future strategies for disease prevention and control in crab farming.

## 2. Materials and Methods

### 2.1. Crabs, Challenge Test, and Sample Collection

Healthy Chinese mitten crabs, weighing approximately 50 g each, without sex selection, were purchased from an aquaculture farm in Weishan Lake (Jining, Shandong, China). The health status of the crabs was assessed based on their external appearance and behavioral characteristics. Only crabs exhibiting intact appendages, normal locomotor activity, and responsive behavior to external stimuli, with no visible signs of injury or disease, were selected for the experiments. The selected crabs were acclimatized for one week at 21 °C before experimentation. During acclimatization and throughout the experiment, crabs were fed twice daily with a commercial pelleted crab feed (Xingchang Aquatic Technology Co., Binzhou, China). Water quality was maintained with dissolved oxygen of 6.69 ± 0.40 mg/L, pH of 6.82 ± 0.45, unionized ammonia below 0.2 mg/L, and nitrite below 0.05 mg/L. Crabs were maintained under freshwater conditions (salinity about 0 ppt). Crabs were randomly assigned to either a control group or an *Aeromonas hydrophila* infection (Ah) group. For the infection group, each crab in the Ah group was administered a 100 μL intramuscular injection of *A. hydrophila* suspension (2 × 10^7^ CFU/mL in PBS) at the base of the fourth pereiopod. In contrast, the control group received an equal volume of sterile PBS. No mortality occurred in either group during the 24 h challenge. At 24 h post-injection, 0.5 mL of hemolymph was collected from the base of the fourth pereiopod of each crab using a sterile syringe and immediately mixed with anticoagulant solution (27 mM sodium citrate, 115 mM glucose, 336 mM NaCl, and 9 mM EDTA; pH adjusted to 7.0) at a 1:1 (*v*/*v*) ratio. Hemolymph from three randomly selected crabs within each group was then pooled to form a single sample (e.g., Control-1). Three independent pooled samples (*n* = 9 crabs per group) were prepared for each treatment. Hemocytes were pelleted by centrifugation (800× *g*, 10 min, 4 °C), resuspended in 1 mL of TRIzol reagent, and stored at −80 °C until RNA extraction. A total of 18 crabs were used for tRF sequencing, and an additional 18 crabs were used for RT-qPCR validation. Identical experimental challenge, hemolymph collection, and sample processing procedures were applied in both cases. The suspension of *A. hydrophila* was prepared as previously described [2].

### 2.2. RNA Extraction and Quality Analysis

Total RNA was isolated using the TRIzol reagent following the manufacturer’s protocol. Briefly, samples were homogenized in TRIzol, incubated for 5 min at room temperature, and centrifuged (12,000× *g*, 5 min, 4 °C). The supernatant was mixed with 200 μL of chloroform, incubated for 5 min, and centrifuged (12,000× *g*, 15 min, 4 °C). The upper aqueous phase was mixed with an equal volume of isopropanol, incubated for 10 min, and centrifuged (12,000× *g*, 10 min, 4 °C) to precipitate RNA. The pellet was washed with 75% ethanol, air-dried, and subsequently dissolved in 20 μL of RNase-free water. The concentration of RNA was quantified fluorometrically using a Qubit 4 fluorometer with the Qubit RNA HS Assay Kit (Thermo Fisher Scientific, Waltham, MA, USA), and RNA integrity was evaluated by electrophoresis on a 1% agarose gel. To further confirm sample quality and suitability for downstream applications, the size distribution and concentration of small RNAs were evaluated using a LabChip microfluidic system (PerkinElmer, Waltham, MA, USA).

### 2.3. tRF Library Construction and Sequencing

For chemical demethylation, 200 ng of total RNA was incubated in a 50 μL reaction mixture containing 50 mM HEPES buffer (pH 8.0, 15630080, Gibco; J63578, Alfa Aesar, Ward Hill, MA, USA), 75 μM FeH_8_N_2_O_8_S_2_ (pH 5.0), 1 mM α-ketoglutarate (K1128-25G, Sigma-Aldrich, St. Louis, MO, USA), 2 mM C_6_H_7_NaO_6_, 50 mg/L bovine serum albumin (A7906-500G, Sigma-Aldrich), 4 μg/mL AlkB (Guangzhou Epibiotek Co., Ltd., Guangzhou, China), and 2000 U RNase inhibitor. The reaction mixture was maintained at 37 °C for 30 min. After demethylation, the RNA was treated with the enzyme T4 polynucleotide kinase (T4 PNK). The 50 μL reaction contained 5 μL of 10× PNK buffer (B0201S, New England Biolabs, Ipswich, MA, USA), 10 mM ATP (P0756S, New England Biolabs), and 10 U T4 PNK (M0201L, New England Biolabs), and was incubated at 37 °C for 20 min. RNA was purified using TRIzol reagent. Purified RNA fragments ranging from 15 to 45 nucleotides (nt) were size-selected by polyacrylamide gel electrophoresis (PAGE) and recovered. Subsequently, adapter ligation and library amplification were performed using the QIAseq^®^ miRNA Library Kit (QIAGEN, Hilden, Germany; Cat. No. 331505). The constructed libraries were subjected to high-throughput sequencing on a SURFSeq 5000 platform (Guangzhou Epibiotek Co., Ltd., Guangzhou, China) using a single-end 100-nt (SE100) configuration.

### 2.4. tRF Annotation and Differential Expression Analysis

tRF sequencing data were annotated using the SPORTS1.1 software, with reads aligned to the tRNA sequences in the Chinese mitten crab genome (ASM2467909v1). Annotation of tRFs was conducted against both precursor tRNA (pre-tRNA) and mature tRNA sequences. The mature tRNA references were generated by excising predicted introns, appending a CCA sequence at the 3′ end, and appending a 5′ guanine residue to histidine tRNA (tRNA^His^). Based on their genomic origin, tRFs were classified into 5′-tRFs (derived from the 5′ ends of pre-tRNAs or mature tRNAs), 3′-tRFs (originating from the 3′ ends of pre-tRNAs), CCA-tRF (derived from the 3′ ends of mature tRNAs), and internal tRFs (originating from internal regions of tRNAs). Expression profiles of tRFs between experimental groups were analyzed using the DESeq2 R package (version 1.44.0). Only tRFs with an average read per million (RPM) > 0.1 were included, and a threshold of |fold change| > 2.0 (|log_2_FC| > 1.0) with a Benjamini–Hochberg (BH) adjusted *p*-value (FDR) < 0.05 was applied to identify significantly DE-tRFs.

### 2.5. Target Gene Predication and Functional Enrichment Analysis of the Top 30 DE-tRFs

Target gene prediction was performed for the top 30 differentially expressed tRFs only. These were selected from the DE-tRFs by applying |log_2_FC| > 1.0 as a prerequisite and then ranking them by ascending adjusted *p*-value (FDR), retaining the 30 tRF’s with the smallest FDR values. The regulatory targets of these 30 tRFs were predicted using miRanda (version 3.3a) and RNAhybrid (version 2.1). For miRanda, prediction parameters were established with a score threshold ≥ 150, and energy threshold ≤ −20 kcal/mol. For RNAhybrid, the criteria were set to minimum free energy ≤ −20 kcal/mol, *p*-value < 0.05. The resulting predicted target genes were subjected to Gene Ontology (GO) annotation across functional categories of Molecular Function (MF), Biological Process (BP), and Cellular Component (CC). Similarly, Kyoto Encyclopedia of Genes and Genomes (KEGG) pathway enrichment analysis was carried out to annotate target genes against the KEGG database. GO and KEGG enrichment analyses were performed using Fisher’s exact test. *p*-values from Fisher’s exact test were adjusted for multiple comparisons using the BH method, and terms with FDR < 0.05 were considered significantly enriched. Terms with a nominal *p* < 0.05 but FDR ≥ 0.05 were regarded as exploratory trends only. Finally, the enriched GO terms and KEGG pathways were illustrated with bubble plots and bar plots.

### 2.6. RT-qPCR Validation of Differentially Expressed tRFs

To validate the sequencing results, four tRFs were randomly selected from the DE-tRFs (|log_2_FC| > 1.0, FDR < 0.05) for RT-qPCR analysis: tRF-Cys-GCA-1 (5′-GGGGGTGTAGCTCAGTGGTAGAGCATTCGACT-3′), tRF-Gly-GCC-1 (5′-GCATCGGTGGTTCAGTGGTAGAATTCTCGCCT-3′), tRF-Ser-GCT-1 (5′-GACGAGGTGGCCGAGTGGTTAAGGCGTTGGACT-3′), and tRF-Val-CAC-1 (5′-GTTTTCGTAGTGTAGTGGTTATCACGTGCGCCTC-3′). The extraction of total RNA from crab hemocytes was performed using TRIzol reagent. Stem-loop reverse-transcription primers targeting the 3′ terminal sequences of each tRF candidate were designed (Table 1). Reverse-transcription was performed using the PrimeScript RT Reagent Kit (TaKaRa, Dalian, China) in a 20 μL reaction containing 4 μL of 5× PrimeScript Buffer, 1 μL of PrimeScript RT Enzyme Mix l, 1 μL of stem-loop primer (or U6 reverse primer), 1 μL of RNA, and RNase-free water to 20 μL. The reaction was incubated at 37 °C for 15 min, 85 °C for 5 s, and then held at 4 °C. The resulting cDNA was diluted 4-fold with nuclease-free water before qPCR. RT-qPCR were conducted using the TB Green^®^ Premix Ex Taq^TM^ II (TaKaRa, Dalian, China) in a 20 μL reaction containing 10 μL of Supermix, 1 μL each of forward and reverse primers, 1 μL of diluted cDNA, and ddH_2_O to 20 μL. The thermal profile was as follows: initial denaturation at 95 °C for 10 s (1 cycle); 40 cycles of 95 °C for 5 s, 60 °C for 30 s, and 70 °C for 20 s, followed by melting curve analysis (95 °C for 0 s, 65 °C for 15 s, then 95 °C with continuous acquisition at a ramp rate of 0.1 °C s). The amplification efficiency of each primer pair was determined from standard curves and ranged from 90–110% (R^2^ > 0.99). U6 snRNA was used as a reference control, as it is commonly used for normalizing small RNA RT-qPCR in crabs and mammals [17,18]. The stability of U6 was evaluate by comparing its raw Ct values between control and infected hemocytes, which showed no significant difference (*p* > 0.05). All reactions were run in technical triplicate on three biological replicates per group, and the mean Ct value of the three technical replicates was used as a single data point for each biological replicate. Relative expression of tRFs was calculated using the 2^−ΔΔCt^ method. Each tRF was analyzed independently between *A. hydrophila* infected group and the control group (*n* = 3 biological replicates per group), using an unpaired two-tailed Student’s *t*-test (GraphPad Prism, version 10.1.2). *p*-value < 0.05 was considered statistically significant.

## 3. Results

### 3.1. Quality Assessment and Basic Characteristics of tRFs

**Figure 1:**
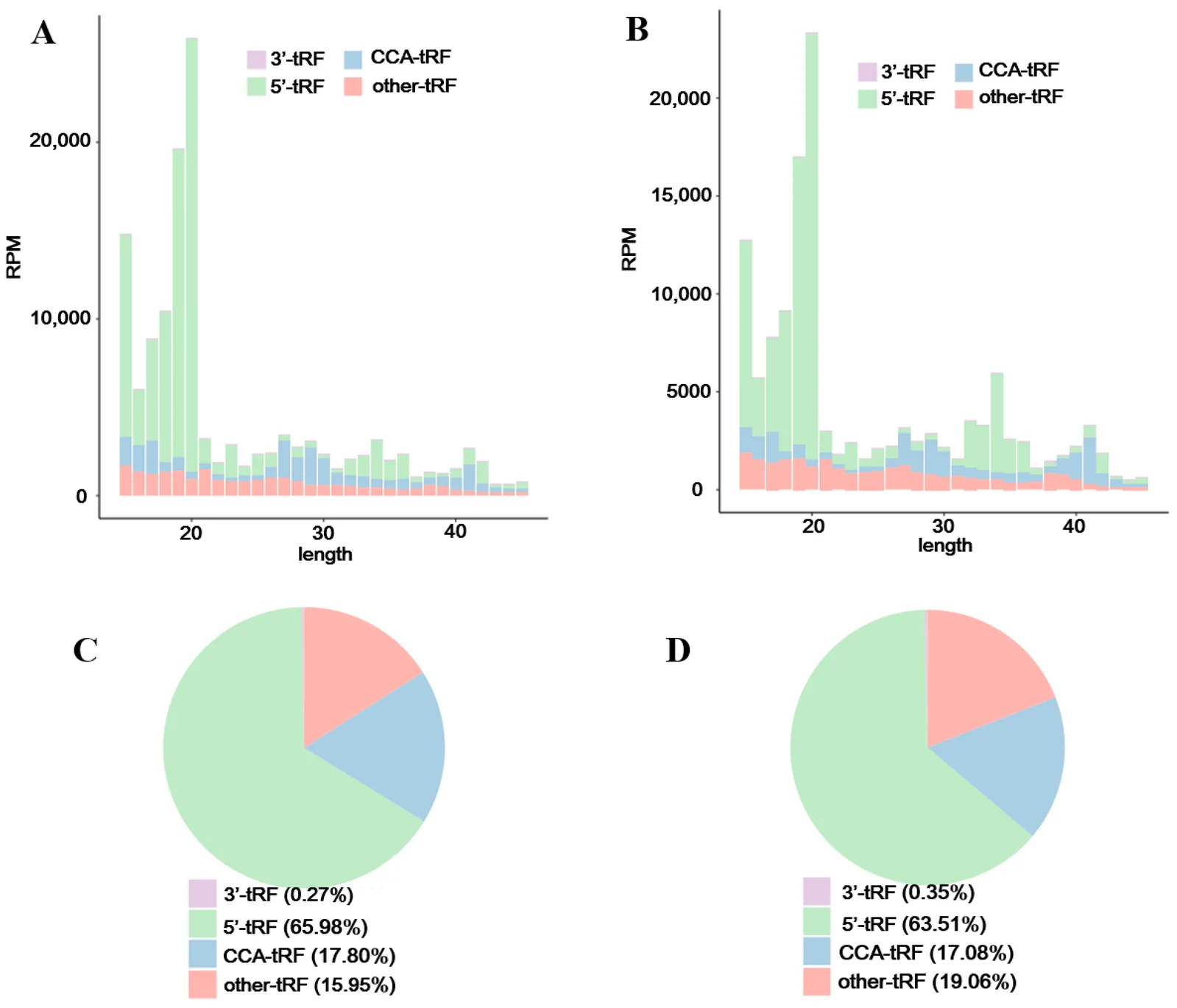
Global characteristics of tRFs in Chinese mitten crab hemocytes. (**A**,**B**) Length distribution and expression abundance (RPM) of tRFs in the control (**A**) and *Aeromonas hydrophila*-infected (Ah) (**B**) groups. (**C**,**D**) Proportional distribution of tRF subtypes in the control (**C**) and *A. hydrophila*-infected (**D**) groups.

After quality control, high-quality clean reads were obtained for all samples (Table 2). The Q30 values exceeded 99.38%, implying a high sequencing accuracy. The mapping rate ranged from 71.56% to 75.13%, and the effective rate exceeded 81.79%, suggesting that most of the reads were reliably assigned to known tRF categories. The basic characteristics of tRFs were analyzed (Figure 1). Expression abundance, normalized as RPM, revealed that a subset of tRFs exhibited relatively high expression levels in both groups. As shown in Figure 1A,B, tRFs in both control and Ah groups displayed a similar length distribution, with the majority of fragments ranging from approximately 15 to 20 nt, indicating a conserved size profile. However, a slight increase in tRFs of approximately 35 nt was observed in the Ah group. Subtype classification revealed that 5′-tRFs (5′-tRF) were the dominant category in both groups, accounting for 65.98% and 63.51% of total tRF in the control and Ah groups, respectively (Figure 1C,D). CCA-tRFs (CCA-tRF) accounted for approximately 17.8% (control group) and 17.08% (Ah group) of the total tRF, while 3′-tRFs accounted for only a small proportion (control: 0.27%; Ah: 0.35%).

### 3.2. Expression Profiles of tRFs in Response to A. hydrophila Infection

**Figure 2:**
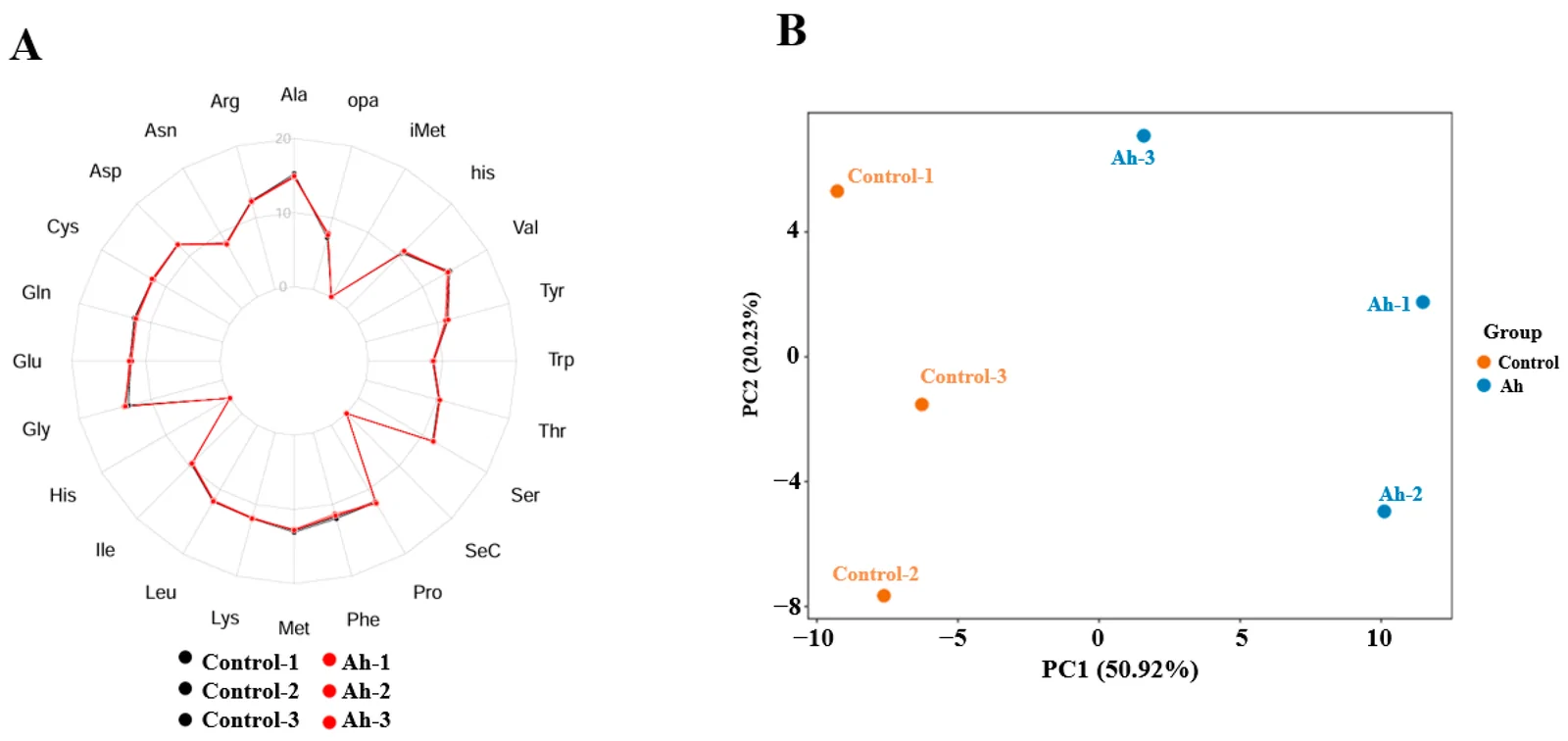
Distribution patterns and sample clustering of tRFs in Chinese mitten crab hemocytes. (**A**) Radar plot illustrating the composition of different tRF subtypes between the control and *A. hydrophila*-infected groups. (**B**) Principal component analysis (PCA) of tRF expression profiles in the control and *A. hydrophila*-infected groups.

To further characterize the distribution patterns of tRF subtypes, a radar plot was generated (Figure 2). The results revealed that the overall composition of tRF subtypes was largely similar between control and Ah groups, although there were minor differences in specific subtypes (Figure 2A). PCA (principal component analysis) was subsequently conducted to evaluate the differences in tRF expression profiles. As illustrated in Figure 2B, samples from the two groups formed distinct clusters, indicating that *A. hydrophila* infection was associated with the distinct separation of tRF expression profiles in crab hemocytes.

### 3.3. Differential Expression of tRFs

**Figure 3:**
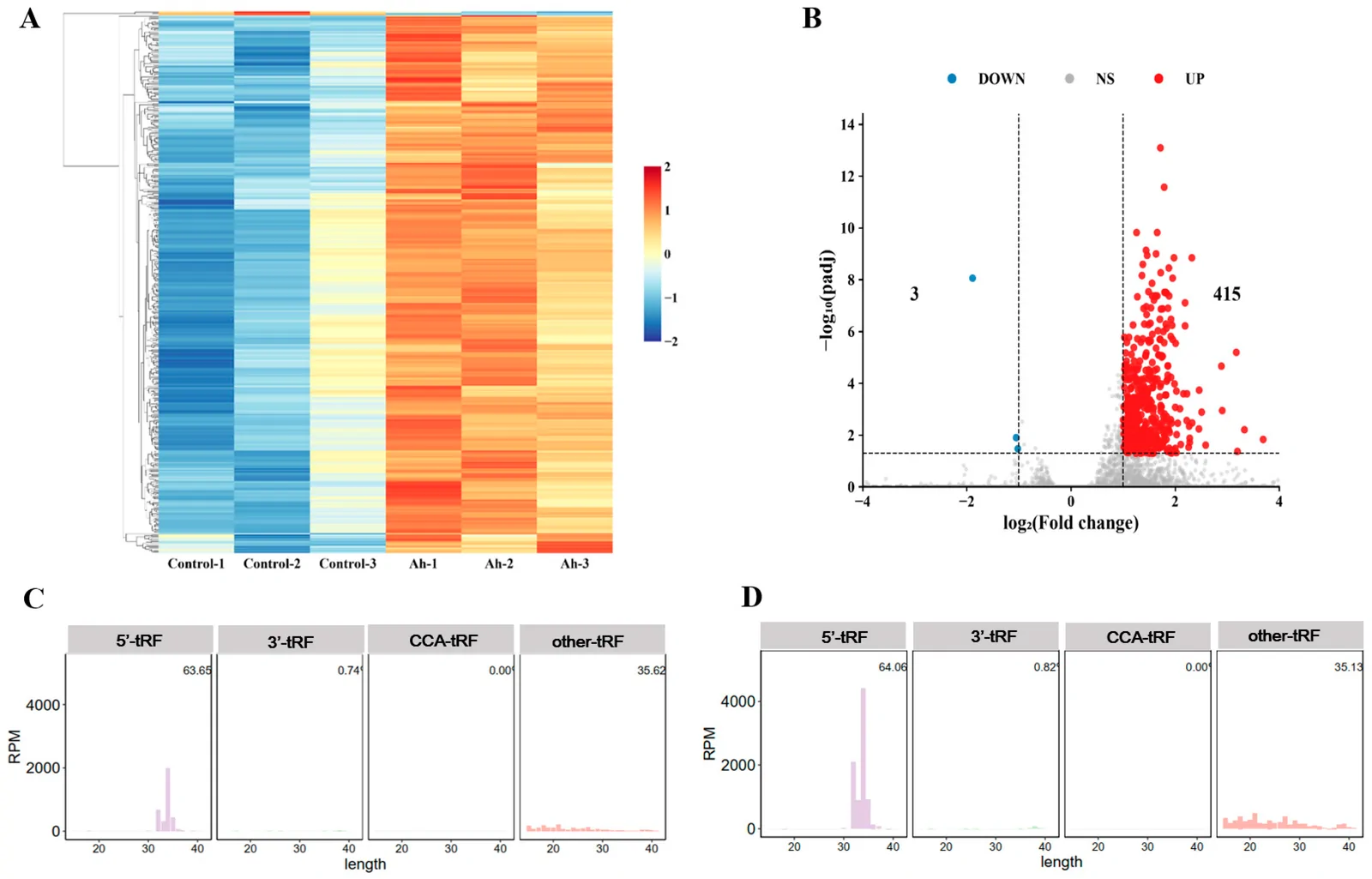
Identification and characterization of differentially expressed tRFs (DE-tRFs) in Chinese mitten crab hemocytes. (**A**) Heatmap showing the expression profiles of DE-tRFs between the control and *A. hydrophila*-infected groups. (**B**) Volcano plot of DE-tRFs between the control and *A. hydrophila*-infected groups. (**C**,**D**) Proportional subtype composition and length of DE-tRFs in the control (**C**) and *A. hydrophila*-infected (**D**) groups.

The results revealed substantial changes in tRF abundance to *A. hydrophila* infection (Figure 3). The hierarchical clustering of the identified DE-tRFs (|log_2_FC| > 1.0, FDR < 0.05) revealed a clear separation of samples within experimental groups, with increased regulation mostly in the Ah group relative to the Control group (Figure 3A). This pattern was confirmed by volcano plot analysis, which identified 415 upregulated and 3 downregulated tRFs (Figure 3B), indicating that infection significantly reconfigures host tRFs.

Size distribution analysis revealed that the DE-tRFs were largely consistent with the global tRF population, except for a marked increase in ~35-nt fragments in the infected group (Figure 3C,D). These fragments were almost exclusively annotated as 5′-tRF, whereas 3′-tRF and CCA-tRF showed no comparable peak, and other-tRF was broadly distributed across 15–40 nt without a distinct peak. In terms of subtype composition, the proportion of 5′-tRF was nearly unchanged between the Ah (64.06%) and Control (63.65%) groups, with a slight decrease in tRF-other (35.13% vs. 35.62%) (Figure 3C,D). These results suggest that *A. hydrophila* infection primarily modulates the abundance of a specific size class within the 5′-tRF subtype rather than altering the overall subtype composition of the expressed tRF population.

### 3.4. GO and KEGG Enrichment of the Top 30 DE-tRFs’ Predicted Target Genes

**Figure 4:**
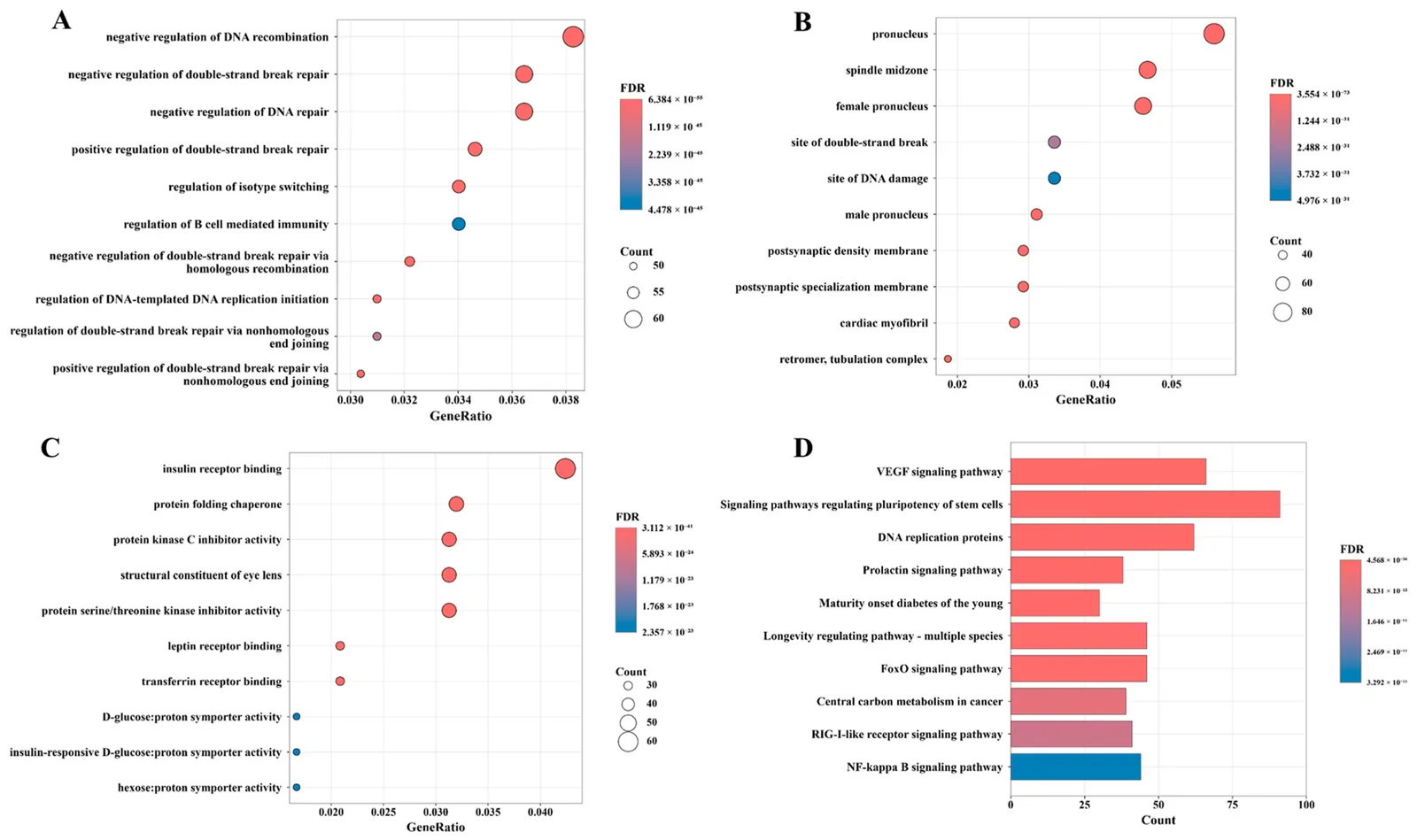
Functional annotation of target genes of the top 30 differentially expressed tRFs (DE-tRFs) in hemocytes of Chinese mitten crab. (**A**–**C**) Gene Ontology (GO) enrichment analysis of predicted targets across the (**A**) Biological Process, (**B**) Cellular Component, and (**C**) Molecular Function domains. (**D**) KEGG pathway enrichment analysis of the predicted target genes.

To investigate the functional relevance of the DE-tRFs, we performed target gene prediction for the top 30 DE-tRFs (selected by |log_2_FC| > 1.0 and the smallest FDR values), followed by functional enrichment analysis (Figure 4). Figure 4 shows the functional annotation of the predicted target genes of the top 30 tRFs. In the GO classification, the predicted target genes were assigned to three main categories: Biological Process, Cellular Component, and Molecular Function. Within the Biological Process category, enriched terms included the negative regulation of DNA recombination, negative regulation of double-strand break repair, negative regulation of DNA repair, and negative regulation of double-strand break repair via homologous recombination (Figure 4A). For the Cellular Component category, enriched annotations included the pronucleus, spindle midzone, side of double-strand break, and site of DNA damage (Figure 4B). In the Molecular Function category, the significantly enriched terms were mainly related to insulin receptor binding, protein folding chaperone activity, protein kinase C inhibitor activity, and protein serine/threonine kinase inhibitor activity (Figure 4C).

KEGG pathway analysis of the predicted target genes assigned the genes to pathways involved in the VEGF signaling pathway, DNA replication proteins, FoxO signaling pathway, and NF-kappa B signaling pathway (Figure 4D). Collectively, these results suggest that these top 30 DE-tRFs may be associated with immune-related processes in crab hemocytes during *A. hydrophila* infection.

### 3.5. RT-qPCR Analysis

**Figure 5:**
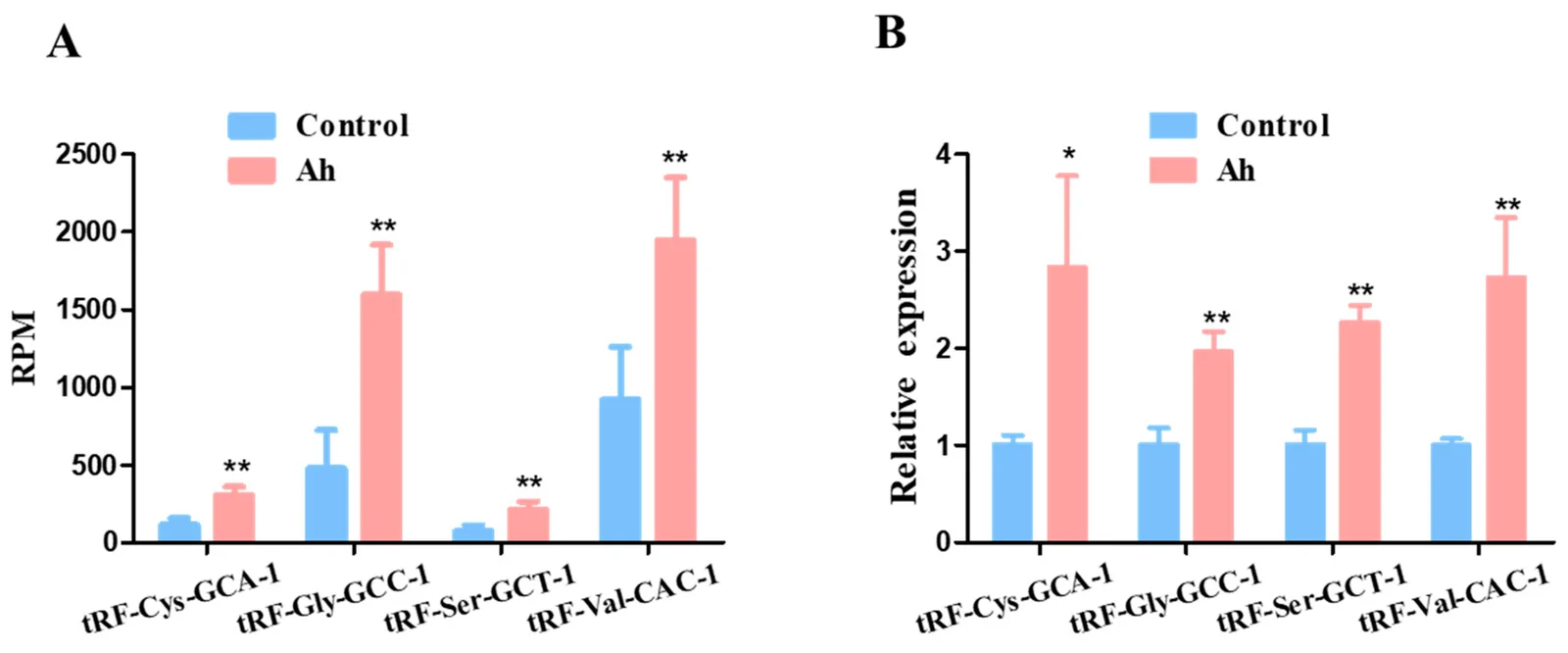
Validation of selected differentially expressed tRFs hemocytes of the Chinese mitten crab, comparing control and *A. hydrophila*-infected groups. (**A**) Expression levels of tRFs from tRF-seq, shown as reads per million (RPM). Bars represent mean ± SD (*n* = 3). Differential expression was determined using DESeq2 (|fold change| > 2.0 and Benjamini–Hochberg adjusted *p*-value (FDR) < 0.05); ** indicates adjusted *p*-value < 0.01. (**B**) RT-qPCR analysis results. Data are presented as mean ± standard deviation (SD) from three independent biological replicates (*n* = 3), with three technical replicates performed for each biological replicate. Error bars represent SD. Statistical significance was assessed using an unpaired two-tailed Student’s *t*-test. * *p* < 0.05; ** *p* < 0.01.

To validate the sequencing results, four DE-tRFs (tRF-Cys-GCA-1, tRF-Gly-GCC-1, tRF-Ser-GCT-1, and tRF-Val-CAC-1) were chosen for further analysis by stem-loop RT-qPCR (Figure 5). Consistent with the sequencing profiles (Figure 5A), all selected tRFs showed a significantly increased expression after *A. hydrophila* infection (Figure 5B). The results confirmed the accuracy and reliability of tRF sequencing data.

## 4. Discussion

tRFs are small non-coding RNAs generated by the targeted cleavage of tRNAs (both mature and precursor forms) under physiological and pathological conditions [19]. Previous studies have established that tRFs participate in diverse biological processes, including translational regulation, gene expression modulation, mRNA stability control, epigenetic modifications, chromatin remodeling, and pathogen–host crosstalk across eukaryotic organisms [20,21,22]. However, compared with well-characterized small RNAs, e.g., small-interfering RNAs, microRNAs, and piwi-interacting RNAs [23,24,25], the immune functions of tRFs in crustaceans remain poorly understood, especially their expression patterns and downstream regulatory roles during bacterial infection. Herein, we conducted a high-throughput sequencing analysis to characterize the expression patterns of tRFs in hemocytes of Chinese mitten crabs following *A. hydrophila* infection. Our results revealed that *A. hydrophila* infection is accompanied by marked changes in the tRF repertoire, which may be related to host innate immune responses and metabolic homeostasis.

Both the uninfected control and infected Ah groups exhibited a predominant tRF length distribution of 15–20 nucleotides (nt), consistent with findings in other species such as African clawed frog (15–22 nt) and mouse (18–22 nt) [26]. The identified tRFs were classified into four major categories, of which 5′-tRFs constituted the predominant subtype, followed by CCA-tRFs, whereas 3′-tRFs accounted for only a minor proportion. The high abundance of 5′-tRFs observed in this study may point to an important contribution of this subtype to the physiological homeostasis of the Chinese mitten crab. Our findings corroborate recent reports showing that pathogenic infection triggers the production of longer tRF fragments and preferential accumulation of 5′-tRFs, which have been described as immune regulators in host defense responses [27]. Similarly, Jiang et al. [28] identified 5′-tRFs as the predominant subtype in the epicardial fat of patients with atrial fibrillation. Although radar plot analysis did not reveal a clear separation between infected and control groups at the tRF expression level, PCA showed distinct clustering between control and Ah groups, suggesting separation in the overall tRF expression profiles following *A. hydrophila* infection. This pattern is consistent with previous findings that a bacterial challenge reshapes the host tRF expression profile [28].

Differential expression analysis, visualized through heatmaps and volcano plots, identified a substantial number of significantly altered tRFs in response to infection, suggesting that tRF abundance changes markedly during the host response to pathogenic stimulation. Specifically, we identified 415 upregulated and 3 downregulated tRFs following *A. hydrophila* infection, revealing a strong bias toward increased tRF abundance. This widespread upregulation pattern aligns with previous studies showing that tRFs are involved in host–pathogen interactions [20], which is compatible with a possible contribution of tRF-associated regulatory networks to crustacean innate immune activation. Similar findings have been reported in humans infected with hepatitis B virus or hepatitis C virus [29], respiratory syncytial virus [30], and SARS-CoV-2 [27]. Collectively, these results suggest that tRF expression reprogramming is a prominent molecular event during hemocyte responses to *A. hydrophila* infection.

Target gene prediction was performed for the selected top 30 DE-tRFs, and the GO and KEGG enrichment analyses was based on the predicted target genes of these top 30 DE-tRFs. The GO enrichment analysis of the predicted target genes of these top 30 DE-tRFs revealed that, within the Biological Process category, the target genes were mainly associated with the negative regulation of DNA recombination, double-strand break repair, and homologous recombination. The terms belonging to the Cellular Component included the pronucleus, spindle midzone, site of double-strand break, and site of DNA damage. Although these terms are related to the cell cycle and genomic stability, their enrichment during bacterial infection raises the possibility that these top 30 DE-tRFs are linked to a stress response program in hemocytes that prioritizes genomic surveillance. Similar DNA damage responses in hemocytes have been reported in the mud crab (*Scylla paramamosain*) following *Vibrio parahaemolyticus* infection [31]. Previous transcriptomic studies on Chinese mitten crabs following *A. hydrophila* infection have mainly focused on typical immune pathways, such as Toll, IMD, and JAK/STAT, without considering the processes involved in DNA repair [32]. Therefore, our findings might extend the understanding of the mechanisms of antibacterial immune regulation in crabs, and tentatively point to a possible link between tRFs and pathways that maintain genomic stability.

The enrichment of Molecular Function terms among the predicted targets of the top 30 DE-tRFs, including insulin receptor binding, protein folding chaperone activity, protein kinase C inhibitor activity, and protein serine/threonine kinase inhibitor activity, is compatible with a potential role of these tRFs in the attenuation of signaling and maintenance of protein homeostasis. Inhibitors of protein kinase C and serine/threonine kinase are known to act as key negative regulators of inflammatory signaling [33], and their enrichment hints that these tRFs could contribute to limiting excessive immune activation. This is consistent with a previous study showing that tsRNAs are involved in host–microbe inflammatory responses and regulate inflammatory mediators [34]. Furthermore, it is known that *A. hydrophila* infection triggers a strong inflammatory response in crabs [32]. Uncontrolled inflammation can affect host survival, and mechanisms that reduce immune intensity are important. Previous studies in mammals have demonstrated that tRFs can target specific transcription factors to modulate inflammatory responses [35], and our findings raise the possibility that a similar regulatory mechanism operates in crustaceans, although this remains to be experimentally confirmed.

KEGG pathway analysis of the predicted targets of the top 30 DE-tRFs identified VEGF signaling, DNA replication proteins, FoxO signaling, and NF-κB signaling as the most significantly enriched pathways. The involvement of VEGF signaling is particularly noteworthy. In Chinese mitten crabs, EsVEGF1 and EsVEGFR4 play a mediating role in bacterial recognition and the regulation of antimicrobial peptide expression [36]. The evolutionary conservation of VEGF signaling during immune cell migration has been demonstrated in *Drosophila*, where the VEGF signaling pathway directs hemocyte migration in inflammatory responses [37]. Our results tentatively suggest that some of the top 30 DE-tRFs may be linked to VEGF signaling during *A. hydrophila* infection, with the potential, though unconfirmed, influence on hemocyte migration and antimicrobial peptide production. The enrichment of FoxO signaling is consistent with its established role in the antibacterial defense mechanism in arthropods. In shrimp, FoxO-mediated antibacterial immunity is coordinated through PDGF/VEGF signaling to maintain hemolymph microbiota homeostasis [38], and the ROS/Aqp4/FoxO/Relish/AMPs axis has been characterized in crayfish [39]. Given that *A. hydrophila* infection causes oxidative stress in crabs, it is conceivable that these tRFs are connected to this ROS–FoxO–AMP regulatory axis, a hypothesis that warrants direct experimental testing. The enrichment of NF-κB signaling is consistent with the role of this pathway in antibacterial immunity in crustaceans. In Chinese mitten crabs, MALT1 promotes antibacterial responses by activating NF-κB signaling and enhancing the phagocytic ability of hemocytes [40]. Our results point to a possible association between these DE-tRFs and this pathway during *A. hydrophila* infection, which needs to be verified experimentally.

Based on target prediction and the enrichment of the top 30 DE-tRFs, these tRFs appear to be potentially associated with multiple regulatory processes, including genomic stability maintenance, signaling attenuation, and immune pathway modulation, which could collectively contribute to the antibacterial immune response of the Chinese mitten crab against *A. hydrophila* infection. The expression patterns of four selected tRFs, validated by stem-loop RT-qPCR, were highly consistent with the sequencing data, confirming the high reproducibility of our high-throughput sequencing result. This concordance is in agreement with earlier reports [25,26].

## 5. Conclusions

This study provides a comprehensive profile of tRFs in the Chinese mitten crab infected with *A. hydrophila*. Our findings suggest that bacterial infection induces specific tRF reprogramming, and the bioinformatics target prediction and enrichment analysis of the top 30 DE-tRFs indicate possible associations with immune signaling pathways. Our work broadens the current view of non-coding RNA-mediated innate immunity in crustaceans and identifies potential tRF candidates for future antibacterial research. However, as the precise molecular targets and functional mechanisms of the key DE-tRFs remain to be elucidated, further gene overexpression and interference experiments are required to elucidate their roles in host–pathogen interactions.

## Figures and Tables

**Table 1:** Primers used for stem-loop RT-qPCR validation of tRFs in the Chinese mitten crab (*Eriocheir sinensis*).

Primer	Sequence (5′-3′)	Objective
R	CTCAACTGGTGTCGTGGA	RT-qPCR
Cys-RT	CTCAACTGGTGTCGTGGAGTCGGCAATTCAGTTGAGCAGTCGAAT	RT-qPCR
Cys-F	TCGGCAGGGGGGGTGTAGCTCA	RT-qPCR
Gly-RT	CTCAACTGGTGTCGTGGAGTCGGCAATTCAGTTGAGCAGGCGAGA	RT-qPCR
GlyF	TCGGCAGGGCATCGGTGGTTCA	RT-qPCR
Ser-RT	CTCAACTGGTGTCGTGGAGTCGGCAATTCAGTTGAGCAGTCCAAC	RT-qPCR
Ser-F	TCGGCAGGGACGAGGTGGCCGA	RT-qPCR
Val-RT	CTCAACTGGTGTCGTGGAGTCGGCAATTCAGTTGAGCGAGGCGCA	RT-qPCR
Val-F	TCGGCAGGGTTTTCGTAGTGT	RT-qPCR
U6_F	CTCGCTTCGGCAGCACA	RT-qPCR
U6_R	AACGCTTCACGAATTTGCGT	RT-qPCR

**Table 2:** Quality assessment and annotation statistics of tRFs sequencing data from hemocytes of the Chinese mitten crab.

Sample Name	Raw Reads	Clean Reads	Input Reads for SPORTS	Q30 (%)	Effective Rate (%) *	Mapping Rate (%) **
Control-1	50,987,405	42,592,514	23,918,817	99.52	83.54	71.56
Control-2	53,156,766	43,856,104	23,669,659	99.49	82.50	73.60
Control-3	52,209,553	42,699,672	23,215,210	99.50	81.79	72.60
Ah-1	62,628,266	52,682,051	29,736,536	99.47	84.12	75.00
Ah-2	49,711,197	41,481,405	22,486,010	99.44	83.44	75.13
Ah-3	64,929,112	54,030,536	31,796,645	99.38	83.21	74.61

* Mapping rate (%) = Reads mapped to the genome/Input reads for SPORTS × 100; ** Effective rate (%) = Clean reads/Raw reads × 100.

## Data Availability

The sequencing data generated in this study have been deposited in the NCBI Sequence Read Archive (SRA) database under the accession number PRJNA1502377.
